# Implementing Effective, Evidence-Based Older Adult Fall Prevention

**DOI:** 10.1093/geroni/igaa057.2796

**Published:** 2020-12-16

**Authors:** Gwen Bergen

## Abstract

Over one in four older adults (65 years and older) in the US reports falling annually with estimated medical costs of $50 billion. Evidence-based strategies exist that can reduce falls with one of the most promising being multifactorial, clinically-based initiatives such as the Centers for Disease Control and Prevention’s STEADI (Stopping Elderly Accidents, Deaths, and Injuries) Initiative. STEADI includes three core components for health care providers: screen for risk factors, assess modifiable factors, and intervene to reduce falls with evidence-based strategies. Barriers to implementation include competing patient demands and limited time during patient visits. Efficient, effective implementation of clinical fall prevention is important to increase the use of multifactorial interventions. In addition, understanding older adult attitudes about the preventability of falls is needed to increase patient adherence to prescribed interventions. This symposium will cover:1. Background data on older adult falls over time,2. Description of an initial implementation of STEADI in an outpatient, Southeastern clinical practice including lessons learned,3. Attitudes of older adults toward fall prevention with implications for health promotion,4. Process evaluation of an ongoing implementation of STEADI in New York State with lessons learned. Understanding practical methods of implementing the three core components of fall prevention into practice supports wider dissemination of evidence-based fall prevention, while understanding patient attitudes toward falls informs the design of health promotion approaches to increase patient uptake of prescribed interventions. Wider dissemination and increased patient adherence in combination can reduce older adult falls and their associated medical costs.

